# Recurrent asymptomatic large pleural effusion due to endometriosis. A case report

**DOI:** 10.1016/j.rmcr.2023.101877

**Published:** 2023-06-03

**Authors:** Abdelrahman Elsayed, Mohamed Elmarasi, Abdulrahman Hamad, Mhd Baraa Habib

**Affiliations:** a College of Medicine, Qatar University, Doha, Qatar; b Department of Internal Medicine, Hamad Medical Corporation, Doha, Qatar

**Keywords:** Pleural effusion, Hemothorax, Endometriosis, Asymptomatic, Catamenial

## Abstract

Pleural endometriosis is a rare manifestation of endometriosis that usually presents with catamenial symptoms, with or without complications. Here, we present a case of incidentally discovered pleural involvement of endometriosis in an asymptomatic young female. Pleurocentesis revealed bloody exudative pleural effusion with lymphocytic predominance. Thoracoscopy revealed inflamed parietal pleura, and the biopsy confirmed endometriotic involvement.

## 1 Introduction

Pleural fluid accumulation is a common consequence of over fifty multiple diseases. Isolated pleural effusion is a diagnostic challenge as it is commonly associated with a series of conditions such as trauma, mycobacterium tuberculosis, and metastatic or local malignancy.

Thoracic endometriosis (TE) is a rare manifestation of endometriosis. TE refers to the presence of endometrial glands in the lungs, pleura, or diaphragm. Although the thorax is the most common site of extra-abdominopelvic endometriosis, its prevalence is not well reported and is probably underdiagnosed [1].

With the presence of endometrial glands in the thoracic cavity, it is not unusual to have recurrent catamenial symptoms of chest pain, shortness of breath, recurrent pneumothorax, hemothorax, and hemoptysis as reported in many case reports in the literature [2]. On the contrary, very few cases were reported with incidental exudative pleural effusion due to endometriosis [3,4].

In this case report we are presenting a rare case of TE with an atypical presentation highlighting the importance of a comprehensive approach to patients presenting with pleural effusions and reviewing prior cases reported with asymptomatic endometriosis-related effusions.

## 2 Case description

**Fig. 1:**
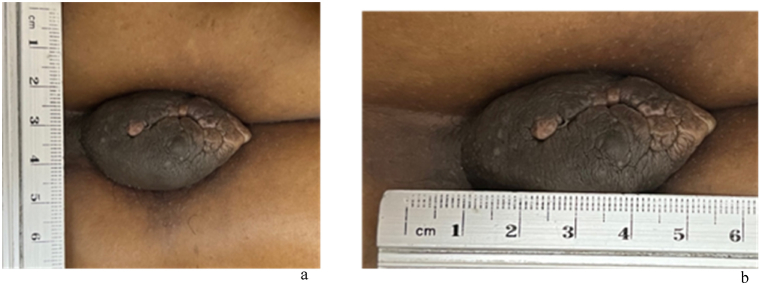
Soft mobile non-tender umbilical mass proven by histopathology to be endometriosis related.

A 31-year-old Nigerian woman with a history of endometriosis and primary infertility was referred to the hospital after an abnormal chest X-ray, which was performed for immigration purposes, showed a large right-sided pleural effusion. Despite having no respiratory or constitutional symptoms, an examination revealed dullness to percussion and absent breath sounds on the right lower and middle lung zones. The patient had a history of endometriosis four years prior, which was characterized by severe dysmenorrhea. Hormonal therapy was prescribed for three months, and an excisional biopsy was taken from an umbilical mass (Fig. 1) that occasionally bled during menses. The mass was later proven to be of endometrial origin.

**Fig. 2:**
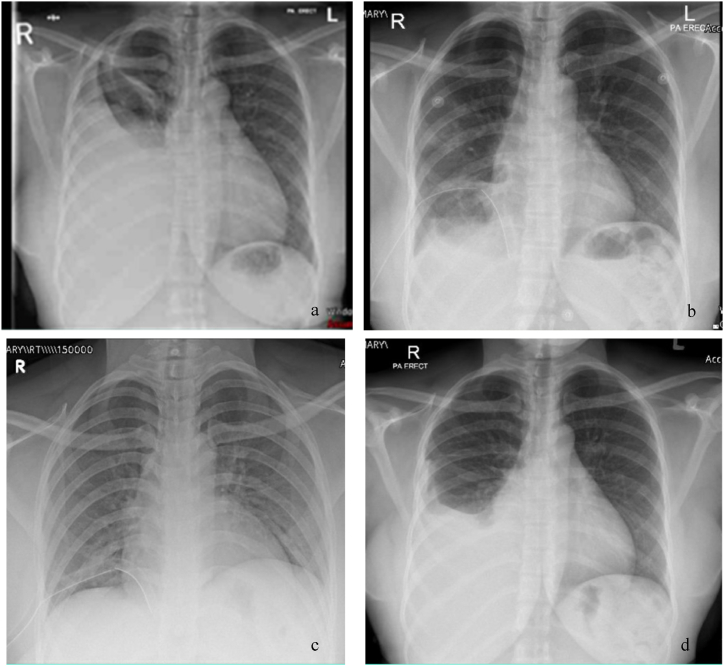
a. CXR at the time of initial presentation showing massive right sided pleural effusion in the upper and middle right lung zones. b. CXR done after thoracoscope. c. The last CXR before discharge. d. CXR done after one month of discharge by the time the patient had menstruation demonstrating re-accumulation of pleural effusion.

**Fig. 3:**
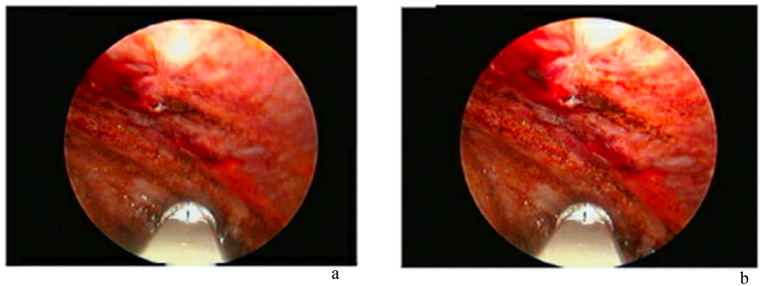
Thoracoscopic inspection of the parietal pleura revealing inflammation.

Upon admission, a complete blood count was unremarkable except for mildly reduced hemoglobin and low albumin. C-reactive protein was normal, and initial work-up for *Mycobacterium tuberculosis* was negative. A chest X-ray (Fig. 2a) revealed pleural effusion, and pleural tapping yielded bloody fluid that was sent for analysis and microscopy. The analysis revealed glucose of 5.3 mmol/L, LDH 314 U/L, and protein of 47.7 gm/L. The total nucleated cells were 250/μL, and red blood cells (RBCs) were 104,062/μL (Table 1). Thoracoscopy showed extensively inflamed parietal pleura (Fig. 3), and a large bore chest tube was placed afterward. The tissue biopsy confirmed endometriosis.

**Table 1:** Patient initial laboratory results upon presentation including general hematology, blood chemistry and pleural fluid analysis and chemistry.

General hematology	Value w/Units	Normal Range
*WBC*	*8.2* × *1^3^/uL*	*4.0–10.0*
*RBC*	*4.3* × *1^6^/uL*	*3.8-4.8*
*Hgb*	*11.*8 gm/dL	*12.0–15.0*
*Hct*	*38.0%*	*36.0–46.0*
*Platelet*	*276* × *1^3^/uL*	*150–410*
*Absolute Neutrophil count*	*3.8* × *1^3^/uL*	*2.0–7.0*
*Lymphocyte Auto #*	*3.3* × *1^3^/uL*	*1.0–3.0*
*Monocyte Auto #*	*0.8* × *1^3^/uL*	*0.2*–*1.0*
*Eosinophil Auto #*	*0.20* × *1^3^/uL*	*0.02*–*0.50*
*Basophil Auto #*	*0.03* × *1^3^/uL*	*0.02*–*0.10*
*Neutrophil Auto %*	*46.3%*	
*Lymphocyte Auto %*	*40.5%*	
*Monocyte Auto %*	*10.1%*	
*Eosinophil Auto %*	*2.7%*	
*Basophil Auto %*	*0.4%*	
***Blood chemistry***	***Value w/Units***	***Normal Range***
*LDH*	*206 U/L*	*135–214*
*Urea*	*2.*8 mmol/L	*2.5–7.8*
*Creatinine*	*57 μmol/L*	*44–80*
*Sodium*	138 mmol/L	*133–146*
*Potassium*	*3.*8 mmol/L	*3.5–5.3*
*Chloride*	106 mmol/L	*95–108*
*Bicarbonate*	24 mmol/L	*22–29*
*Bilirubin T*	*9 μmol/L*	*0–21*
*Total Protein*	70 gm/L	*60–80*
*Albumin Lvl*	32 gm/L	*35–50*
*Alk Phos*	*48 U/L*	*35–104*
*ALT*	*13 U/L*	*0–33*
*AST*	*18 U/L*	*0–32*
*CRP*	*4.*2 mg/L	*0.0–5.0*

***Pleural fluid analysis***	***Value w/Units***
*BF Type Cell Count*	*Pleural*
*Color BF*	*Bloody*
*Appearance BF*	*Bloody*
*Total Nucleated Cell BF*	*250/uL*
*RBC BF*	*104,062/uL*
*Neutrophils BF*	*3.0%*
*Lymphocyte BF*	*68.0%*
*Monocyte BF*	*3.0%*
*Macrophage BF*	*23.0%*
*Mesothelial BF*	*3.0%*
***Pleural fluid chemistry***	***Value w/Units***
*BF Glucose*	*5.*3 mmol/L
*BF LDH*	*314.0 U/L*
*BF Protein*	*47.*7 gm/L
*BF Albumin*	*28.*4 gm/L

**Fig. 4:**
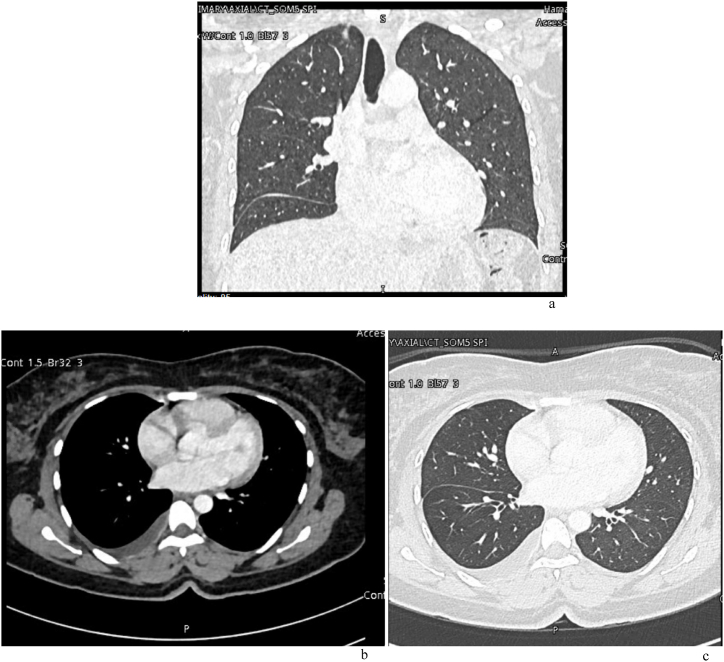
Chest CT scan done after discharge, showing right sided pleural effusion with subtle pleural thickening and bilateral apical thickening.

An abdominal ultrasound revealed fluid in the right and left iliac fossae and a trace of fluid in the subcutaneous fat plane of the irregular umbilical mass. Chest computed tomography (CT) (Fig. 4) showed right pleural effusion with subtle pleural thickening and bilateral apical thickening, as well as mildly enlarged axillary lymph nodes mainly on the right.

**Fig. 5:**
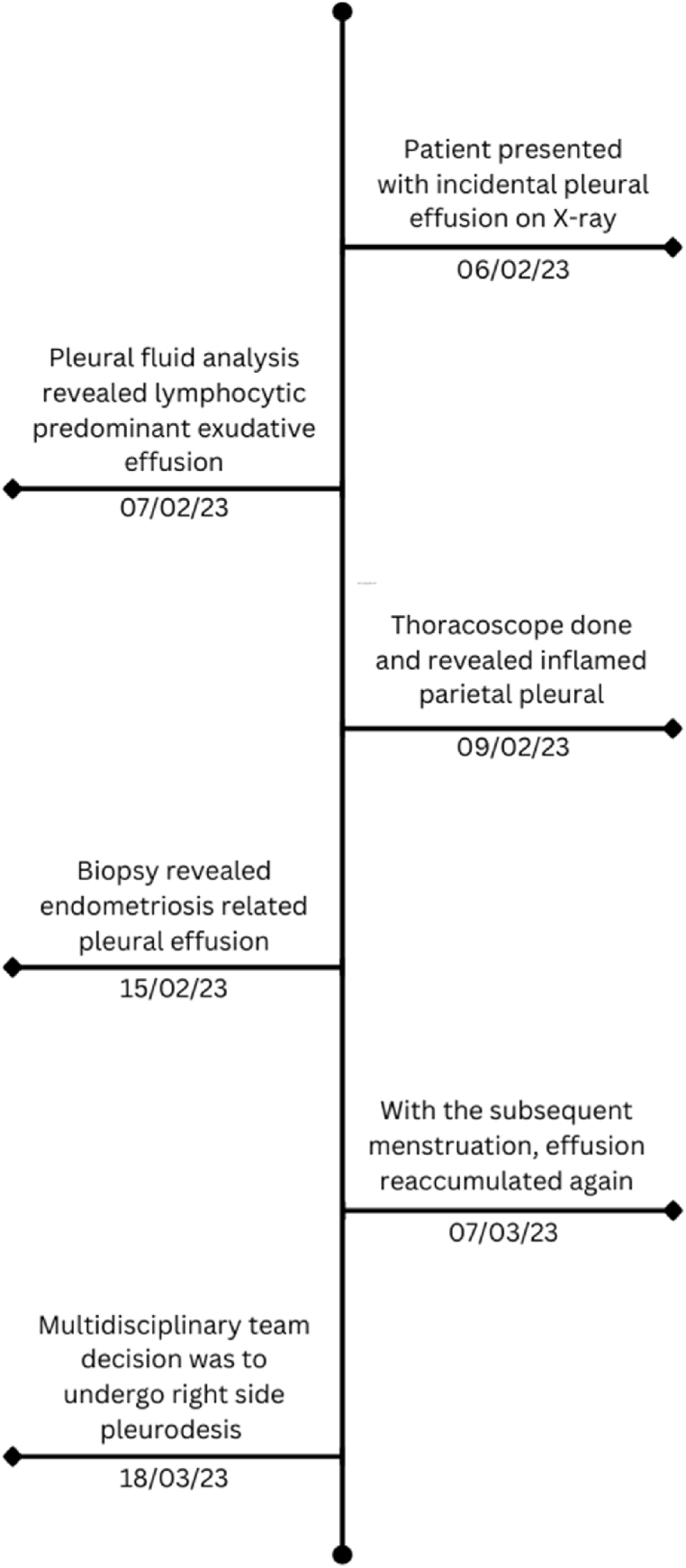
Timeline of events since patient presentation until final treatment of the pleural effusion.

During the patient's follow-up, re-accumulation of pleural fluid was discovered by an X-ray with her menstruation (Fig. 2d). A therapeutic thoracocentesis was done to prevent possible complications of the bloody effusion. After considering the patient's desire for using assisted reproductive technology, a multidisciplinary approach recommended right-side pleurodesis prior to any conception attempts and avoiding hormone suppressive therapy. Fig. 5 demonstrates the timeline order of events from the initial evaluation until the final treatment.

## 3 Discussion

Endometriosis, defined as the presence of endometrial glands outside the uterine cavity, is a well-described chronic disease. Its estimated prevalence among women in the reproductive age group is approximately 10% [5]. Unlike pelvic endometriosis, which has multiple risk factors, the risk factors for thoracic endometriosis are not well known, although it has been associated with infertility and uterine surgeries [6]. After a thorough review of PubMed, Scopus, and Embase, only two cases of pleural endometriosis were found to have totally asymptomatic presentation. Table 2 provides summaries for these two cases.^3,4^ The two cases were of two 30- and 39-year-old women who were discovered by Chest X-ray incidentally as our patient. X-rays showed effusions with air-fluid levels and were described as hydropneumothorax. Therefore, as per our literature review our case is the only case presented with a pure pleural effusion picture and no associated symptoms.

**Table 2:** Literature review. This table presents the only two cases reported in the literature to have asymptomatic pleural involvement due to endometriosis.

References	Present age, Ethnicity	Diagnosis	Findings on X-ray	Past medical/surgical history	Management	Histopathology
**3**	30, African.	X-ray done due to occupational health screening.	X-ray showed right hydropneumothorax. CT thorax demonstrated large right hydropneumothorax occupying approximately 20% of right hemithorax and 1 cm right pleural nodule.	Partial hysterectomy with left unilateral oophorectomy.	Chest tube was inserted draining blood-stained fluid, which came back exudative. Pleural biopsy was taken.	showed benign endometrial glands and chronic inflammation. No malignancy, granulomatous or infectious inflammation was identified.
**4**	39, NA.	X-ray done as for preemployment requirement.	Nearly 100% right-sided pneumothorax with air/fluid levels indicating a hydropneumothorax.	Endometriosis Hypertension Latent tb treated with 9m with INH.	Rt thoracotomy, total pulmonary decortication, visceral and parietal pleurectomy and talc and mechanical pleurodesis.	Showed pleura with scattered deposits of endometriosis with associated acute and chronic pleuritis and mesothelial hyperplasia.

Although pleural endometriosis is a rare complication of the disease, the time interval between the accumulation of fluid and the patients' presentation is not well known, and the interval may vary from one patient to another. In our case, the patient developed pleural effusion despite not having any gynecologic symptoms of endometriosis, raising concerns that the process of pleural involvement can be independent of other aspects of the disease. Later presentation of pleural endometriosis has been reported in many case reports [7], where patients present with respiratory symptoms related to their menstrual cycle and in many circumstances can be complicated by pneumothorax and/or effusions that occur mainly with menstruation, which can be catastrophic for patients [8].

Implication of routine X-ray during the initial time of diagnosis and subsequent follow-ups is not a well-known recommendation in the literature, as the incidence of thoracic endometriosis is perceived as rare.

## 4 Conclusion

Women with endometriosis may develop pleural effusion, even if they do not experience any respiratory symptoms or symptoms related to endometriosis. To avoid complications associated with this condition, we recommend that these patients undergo chest X-ray at the time of initial diagnosis and probably during follow-up visits, also it might be beneficial to rule out pleural involvement in infertile women with endometriosis who might want to try assisted reproductive technologies, which may include therapies that increase the activity of the ectopic endometrial glands. This proactive approach can help identify pleural involvement early and prevent any potential negative impacts on the patient's health.

## Patient perspective

Written consent was taken from the patient.

## Declaration of competing interest

This case report has not been published previously or is not under consideration in another journal. The publication of this work is approved by all the mentioned authors, who have no conflict of interest.
